# Respiratory Distress Syndrome in Infants Delivered via Cesarean from Mothers with Preterm Premature Rupture of Membranes: A Propensity Score Analysis

**DOI:** 10.1155/2020/5658327

**Published:** 2020-07-31

**Authors:** Mariko Nakahara, Shunji Goto, Eiji Kato, Atsuo Itakura, Satoru Takeda

**Affiliations:** ^1^Department of Obstetrics and Gynecology, Juntendo University Faculty of Medicine, Bunkyo-ku, Tokyo, Japan; ^2^Perinatal Center for Maternity and Neonate, Japan Community Health Care Organization Funabashi Central Hospital, Funabashi, Chiba, Japan

## Abstract

**Objective:**

This study aimed to clarify the effects of cesarean delivery on neonatal respiratory morbidity when women had preterm premature rupture of membranes.

**Methods:**

This retrospective study included women with preterm premature rupture of membranes who delivered from 23 weeks to 33 weeks of gestation between January 2009 and December 2014. Neonatal outcomes were compared between infants delivered by cesarean section and those delivered vaginally. The primary outcome was respiratory distress syndrome (RDS). Neonatal intubation and mechanical ventilation periods were secondary outcomes. Propensity score matching was used to compare outcomes between cesarean and vaginal delivery cases.

**Results:**

There were 101 cesarean deliveries and 89 vaginal deliveries. A comparison of the presence or absence of neonatal complications based on the delivery type indicated a higher occurrence of RDS with cesarean deliveries (*P* = 0.025). The intubation and mechanical ventilation periods were not significantly longer in neonates delivered via cesarean section.

**Conclusions:**

Cesarean delivery is a risk factor for neonatal RDS in women with preterm premature rupture of membranes. Trials identifying long-term neonatal prognoses are needed to further develop optimal management strategies in such cases.

## 1. Introduction

Preterm premature rupture of membranes (PPROM) occurs in 3% of all pregnancies [1]. Respiratory distress syndrome (RDS) is reportedly the most common and serious complication following preterm birth [2], and it is known to be a risk factor for cerebral palsy [3]. Zanardo et al. [4] reported that RDS occurred in 55.4% of newborns born to women with PPROM. Cesarean delivery (CD) is a risk factor for RDS in preterm neonates; however, other risk factors exist, such as low birth weight [5], maternal multiparity, male sex [6], and prepregnancy body mass index (BMI) [7].

Treatment with prenatal corticosteroids, the onset of labor, and histological chorioamnionitis have been shown to decrease the incidence of RDS [8–11]. Although a randomized trial would be the ideal research method for determining whether CD is a risk factor for RDS, such a trial would require the selection of optimal cases of CD based on clinical practice. Therefore, a randomized trial is not possible. However, propensity scores, introduced by Rosenbaum and Rubin in 1983, could be used to circumvent these covariate restrictions [12]. This score could be used to create groups that differ according to treatment exposure but have otherwise similar baseline characteristics, thereby allowing randomization [13]. In this study, we performed propensity score matching analyses with the aim of clarifying the effects of CD on neonatal respiratory morbidity.

## 2. Materials and Methods

This retrospective study included singleton pregnancies with PPROM delivered between weeks 24 and 33 of gestation, from January 2009 to December 2014, at Japan Community Healthcare Organization Funabashi Central Hospital. Those with maternal malignancies and fetal chromosome abnormalities were excluded. The Institutional Review Committee of Japan Community Healthcare Organization Funabashi Central Hospital approved this study (March 22, 2018; no. H 30-5). Informed consent was obtained from all patients. This study was conducted in accordance with the Declaration of Helsinki.

Neonatal outcomes were compared based on the type of delivery (CD or vaginal). The primary outcome was the occurrence of RDS, which was defined as the number of neonates who developed RDS. Secondary outcomes were neonatal intubation and mechanical ventilation. PROM (premature rupture of membranes) was defined as amniotic fluid leakage (defined as classical PROM) or a clear positive immunochromatographic dipstick test for insulin-like growth factor-binding protein [1]. Before analyzing amniotic fluid secreted before the onset of labor, a high PROM threshold value was set at 25 *μ*g/L (sensitivity, 95.2%; specificity, 90.5%) [14].

Our PPROM strategy, which was expectant management until 35 weeks, included vital sign and laboratory data monitoring for infection or inflammation. If a patient was not experiencing labor, then our management included 12 mg dexamethasone every 24 h for up to 2 days to improve fetal lung maturity. In cases of spontaneous onset of labor, nonreassuring fetal status, suspected abruptio placentae, or clinical chorioamnionitis, delivery was started immediately. CD was performed, especially in cases of a nonreassuring fetal status and suspected abruptio placentae. For clinical chorioamnionitis, if spontaneous delivery with labor was not likely to occur within a short period of time, then CD was performed. Clinical chorioamnionitis was diagnosed based on maternal fever, blood test results, presence of discharge, abdominal pain, and fetal cardiography results (Lencki criteria) [15].

Peripartum maternal factors, delivery information, and neonatal outcomes were extracted from medical records. Maternal prepregnancy BMI was calculated from the prepregnancy weight and height as follows: BMI = weight (kg)/height (m^2^). Body temperature was measured in degrees Celsius. Maternal and fetal well-being were frequently observed until delivery, and venous blood examinations, including white blood cell counts and C-reactive protein (CRP) levels, were performed every few days. Patients whose last examination was conducted within 24 h before parturition were included in this study. Labor was defined as painful contractions occurring more frequently and regularly than once every 5 min, as confirmed subjectively or with cardiotocography; this information was extracted from the medical records. Fetal distress was defined as a significant abnormality in the fetal heart rate. The PROM latency period was defined as the number of days from membrane rupture to delivery. Maternal diabetes was divided into gestational diabetes mellitus (GDM) and overt diabetes. GDM was determined by performing a 75-g oral glucose tolerance test (OGTT). The diagnostic criteria for GDM is a fasting plasma glucose (FPG) >92 mg/dl and/or 1-h plasma glucose 180 mg/dl, or a 2-h plasma glucose >153 mg/dl. Criteria for overt diabetes is an FPG ≥126 mg/dl, HbA1C ≥6.5%, or a random plasma glucose ≥200 mg/dl. One of these criteria must be met to identify the patient as having overt diabetes in pregnancy [16]. The patients who delivered within the therapeutic window for antenatal corticosteroids were defined as women who delivered within 2-7 days of receiving the initial dose [17].

The delivery route was classified as vaginal delivery or CD. Diagnosis of RDS was based on characteristic clinical signs, such as tachypnea, retractions, nasal flaring, grunting, cyanosis, and chest X-ray findings of a reticulogranular pattern. Severe neonatal morbidity was defined as intraventricular hemorrhage [18] or cystic periventricular leukomalacia (i.e., periventricular white matter echolucencies on ultrasonography) [19]. Neonatal sepsis was defined as culture-proven sepsis.

Statistical analyses were performed using R version 3.4.1 (R Foundation, Vienna, Austria). Differences were considered statistically significant at a confidence level of *P* < 0.05 (two-sided alternative hypothesis).

Demographic and clinical data are presented as frequency distributions and percentages. Continuous variables are expressed as the median and interquartile range. Differences in outcomes between CD and vaginal delivery were calculated using the *χ*2 test or Fisher's exact test for categorical variables, and the *t*-test or Mann-Whitney *U* test for continuous variables. To reduce the effects of treatment selection bias and potential confounding in this observational study, differences in the baseline characteristics of patients were adjusted using propensity score matching. The predicted probability of CD was calculated by running a logistic regression model using all clinically relevant variables. The subject pairs, who delivered via vaginal delivery or CD, were derived using 1 : 1 nearest-neighbor matching with a ±0.05 caliper and no replacement.

After propensity score matching was performed, differences between the two groups were assessed using the paired *t*-test for continuous variables and the McNemar test for categorical variables. Univariate and multivariate logistic regression analyses were performed to determine the risk factors for RDS. The risk factors examined were maternal age, multiparity, prepregnancy BMI, antepartum maternal CRP level, antenatal corticosteroid use, delivery week, and CD.

## 3. Results and Discussion

**Figure 1:**
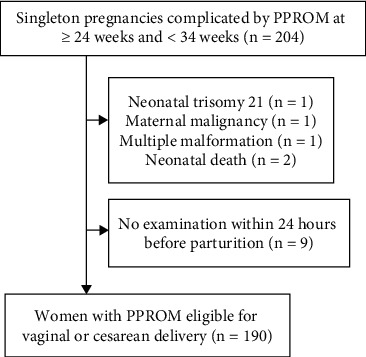
Flow chart of the included participants. PPROM: preterm premature rupture of membranes.

During the study period, 204 singleton pregnancies met our inclusion criteria. Of these, we excluded 14 (fetal trisomy 21, *n* = 1; maternal malignancy, *n* = 1; multiple malformations, *n* = 1; neonatal death, *n* = 2; no examination within 24 h before parturition, *n* = 9). Therefore, 190 women and neonates were analyzed (Figure 1). The median gestational age (GA) at PROM was 30 weeks (interquartile range, 19–33 weeks), and the median GA at delivery was also 30 weeks (interquartile range, 24–33 weeks). Ten women (5.3%) were diagnosed with GDM and 2 (1.0%) were diagnosed with overt diabetes. The number of women who were administered corticosteroids during pregnancy was 103 (54.2%), and the number of women who delivered within the therapeutic window for antenatal corticosteroids was 44 (23.2%).

**Figure 2:**
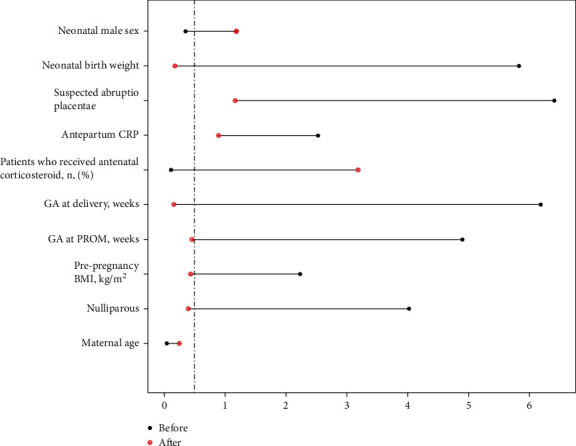
Standardized differences plot before and after propensity score matching.

Matching, using the estimated propensity score, created a matched cohort of 104 patients (52 patients in each group). Patient characteristics before and after matching are listed in Table 1. Prior to matching, there were more multiparous women and the prepregnancy BMI was significantly higher in the vaginal delivery group than in the CD group (*p* = 0.009 and *p* = 0.023, respectively). The GA at PROM and GA at delivery were earlier and the neonatal birth weight was lower in the CD group than in the vaginal delivery group (*p* = 0.001, *p* < 0.001, and *p* < 0.001, respectively). Suspected abruptio placentae occurred more frequently in the CD group than in the vaginal delivery group (*p* < 0.001). However, almost all initially observed differences were balanced in the groups after matching (Figure 2). Neonatal outcomes before and after matching are shown in Table 2. Prior to matching, the incidence of RDS was significantly higher in the CD group than in the vaginal delivery group (*p* < 0.001). The intubation and mechanical ventilation periods were significantly longer in neonates delivered via CD than in those delivered vaginally (*p* < 0.001 and *p* < 0.001, respectively). There were no differences in the incidence of intraventricular hemorrhage between groups (*p* = 0.451). After matching, the incidence of RDS was still higher in neonates delivered via CD than in neonates delivered vaginally; although, the intubation and mechanical ventilation periods were no longer different between the two groups (*p* = 0.025, *p* = 0.144, and *p* = 0.18, respectively).

Our propensity score matching analysis study yielded several important findings. First, we found that delivery via CD increased the risk of RDS in neonates when the mothers had PPROM. A previous meta-analysis also showed that the pooled odds ratio of neonatal RDS associated with CD was 1.76 (95% confidence interval, 1.48–2.09), and both elective CD and emergency CD were associated with an increased risk of neonatal RDS [20]. An epidemiological study on neonatal respiratory diseases in Sweden also reported that acute respiratory morbidity for moderately preterm infants is common and predicted based on multiparity, CD, low Apgar score, and male sex [21]. When determining whether CD increases the risk of RDS, we need to consider the GA, obesity, antenatal corticosteroid use, and any other factors that may affect the occurrence of RDS [6]. In this study, we reduced these biases by using propensity score matching and revealed that CD was still a significant risk factor for RDS.

After performing a propensity score matching analysis, we determined that the mechanical ventilation and intubation periods of newborns delivered by women with PPROM via CD were not significantly longer than those of newborns delivered vaginally. Although mechanical ventilation is a life-saving intervention for premature infants, a longer cumulative duration of mechanical ventilation is associated with increased hospitalization and an increase in the required duration of parenteral nutrition, as well as a higher probability of discharge with poor achievement of physical growth [22]. Although CD should be avoided in women with PPROM to prevent RDS, our study did not show evidence of an association between CD and the duration of ventilation after bias was removed.

This study has a few limitations. The main limitations were that it was nonprospective and nonrandomized. Although a propensity score matching analysis was performed to overcome the weaknesses of retrospective case studies, by reducing bias with estimates resulting from observed differences between CD and vaginal delivery, the study was still subject to bias from unobserved differences. Another limitation was that we could not exclude the effects of labor as a bias, because labor during vaginal delivery was stronger than that during CD. Generally, labor prevents neonatal RDS. The Maternal Fetal Medicine Units Network supports previously published data indicating a higher occurrence of respiratory distress in infants delivered via elective repeat CD than in those delivered via vaginal birth after CD [23]. As hormones such as catecholamines and corticosteroids are produced during labor, the fetal lungs begin producing surfactants. Notably, labor results in the switch from active chloride and fluid secretion to active sodium and fluid absorption in fetal lungs [24]. In this study, whether labor had started was not included as a covariate. Prospective studies that assess factors such as labor timing are needed to reduce bias regarding whether neonatal outcomes change depending on the delivery type (i.e., vaginal delivery or CD) after labor has begun.

In conclusion, CD is a risk factor for neonatal RDS in women with PPROM. Thus, CD should be avoided in women with preterm PROM. Prospective and randomized trials to identify the long-term neonatal prognoses are needed to establish optimal management approaches for PPROM.

## Figures and Tables

**Table 1:** Baseline patient characteristics before and after propensity score matching.

Characteristic	Before matching				After matching			
	Cesarean delivery	Vaginal delivery	*P* value	SMD	Cesarean delivery	Vaginal delivery	*P* value	SMD
Patients, *n*	101	89			51	51		
Maternal age, years	33 [29, 36]	33 [29, 36]	0.904	0.465	33 [31, 36]	34 [30.5, 36]	0.806	2.468
Multiparous	63 (62.4)	38 (42.7)	0.009	40.198	26 (51)	27 (52.9)	1.000	3.925
Prepregnancy BMI, kg/m^2^	19.95 [18.74, 22.36]	21.11 [19.56, 23.80]	0.023	22.295	20.61 [18.85, 22.85]	20.96 [19.52, 23.74]	0.432	4.281
GA at PROM, weeks	29 [26, 31]	30 [28, 32]	0.001	48.997	30 [28, 32]	30 [27, 32]	0.938	4.545
GA at delivery, weeks	30 [27, 31]	32 [30, 32]	<0.001	61.823	30 [29, 32]	30 [28, 32]	0.981	1.546
Patients who received antenatal corticosteroid, *n*, (%)	55 (54.5)	48 (53.9)	1	1.049	24 (47.1)	32 (62.7)	0.163	31.923
Antepartum CRP, mg/dL	1.29 [0.24, 3.00]	0.79 [0.31, 2.14]	0.426	25.252	1.02 [0.38, 2.19]	0.84 [0.21, 2.13]	0.359	8.867
Suspected abruptio placentae, *n* (%)	20 (19.8)	1 (1.1)	<0.001	60.082	2 (3.9)	1 (2.0)	1.000	11.625
Neonatal birth weight, g	1319 [936, 1667]	1692 [1357, 1901]	<0.001	58.239	1523 [1182, 1897.5]	1521 [1199, 1792.5]	0.862	1.72
Neonatal male sex, *n* (%)	55 (54.5)	50 (56.2)	0.884	3.469	30 (58.8)	27 (52.9)	0.690	11.868

Values are presented as numbers (%) or medians [interquartile range]. SMD: standardized mean difference; BMI: body mass index; GA: gestational age; PROM: premature rupture of membranes; CRP: C-reactive protein.

**Table 2:** Neonatal outcomes before and after matching.

Variable	Before matching			After matching		
	Cesarean delivery	Vaginal delivery	*P* value	Cesarean delivery	Vaginal delivery	*P* value
Patients, *n*	101	89		51	51	
RDS, *n*	55 (54.5)	20 (22.5)	<0.001	26 (51.0)	14 (27.5)	0.025
Intubation period, days	2 [0, 21]	0 [0, 1]	<0.001	1 [0, 7]	0 [0, 2]	0.144
Mechanical ventilation period, days	15 [2, 50]	2 [0, 11]	<0.001	5 [1, 29]	1 [0, 29]	0.18
IVH, *n*	5 (5.0)	2 (2.2)	0.451	3 (5.9)	2 (3.9)	0.678
Neonatal sepsis, *n*	4 (4.0)	5 (5.6)	0.735	2 (3.9)	3 (5.9)	1.000

Values are presented as numbers (%) or medians [interquartile range]. RDS, respiratory distress syndrome; IVH, intraventricular hemorrhage.

## Data Availability

The data used to support the results of this study are available from the corresponding author upon reasonable request in order to protect patients' personal data.
